# Age-period-cohort analysis of stroke mortality attributable to high systolic blood pressure in China and Japan

**DOI:** 10.1038/s41598-021-98072-y

**Published:** 2021-09-27

**Authors:** Jinhong Cao, Ehab S. Eshak, Keyang Liu, Ahmed Arafa, Haytham A. Sheerah, Chuanhua Yu

**Affiliations:** 1 Department of Epidemiology and Biostatistics, School of Health Sciences, Wuhan University, 185 Donghu Rd, Wuhan, 430071 Hubei China; 2 Department of Public Health, Community and Preventive Medicine, Faculty of Medicine, Minia University, Minia, Egypt; 3 Present Address: Public Health, Department of Social Medicine, Graduate School of Medicine, Osaka University, Osaka, Japan; 4 School of Public Health, Peking University Health Science Center, Beijing, China; 5 Department of Public Health, Faculty of Medicine, Beni-Suef University, Beni Suef, Egypt

**Keywords:** Hypertension, Preventive medicine, Risk factors

## Abstract

Stroke is a principal cause of mortality in China and Japan. High systolic blood pressure (SBP) was considered a chief risk factor for stroke mortality. Herein, we evaluated temporal trends of high SBP-attributable stroke mortality in China and Japan between 1990 and 2017. Data on stroke mortality were retrieved from the Global Burden of Disease Study 2017 (GBD 2017). Using the age-period-cohort method, we computed overall net drifts, local drifts, longitudinal age curves, and cohort/period rate ratios (RRs) for high SBP-attributable stroke mortality. The age-standardized mortality rates (ASMRs) displayed decreasing trends for high SBP-attributable stroke mortality. The annual net drift values were − 1.4% and − 3.5% in Chinese men and women versus − 3.1% and − 4.9% in Japanese men and women. The local drift values in both countries were < 0 among all age groups but were lower in women than in men. The longitudinal age curves showed a greater high SBP-attributable stroke mortality in men than in women across all age groups. Similar decreasing patterns were shown in the period and cohort RRs in both sexes with women having a quicker decline than men. In China and Japan, the ASMRs, as well as the period and cohort RRs of high SBP-attributable stroke mortality, decreased between 1990 and 2017 in both sexes and across all age groups. Yet, the prevalence of high SBP remained worrisome in both countries. Thus, SBP control should be encouraged to prevent stroke mortality.

## Introduction

High SBP has been, since 2010, at the top of the list of risk factors contributing to the global disease burden^1,2^. It is considered, in particular, a chief risk factor for most cardiovascular diseases including stroke, and even a more important predictor than diastolic blood pressure, mean arterial pressure, and pulse pressure^3–5^. Previous reports showed that up to 60% of stroke mortality worldwide could be related to high SBP of at least 110–115 mmHg while blood pressure control was shown to be the most effective intervention for stroke prevention^6–10^. Biologically, high SBP can contribute to the increased risk of stroke mortality via inducing smooth muscle hypertrophy and remodeling that result in arterial wall thickness and lumen narrowing, enhancing endothelial dysfunction which increases vascular permeability that leads to local thrombi, and accelerating the atherosclerosis process in the intracerebral arteries via enhancing oxidative stress and inflammation that ends with vascular resistance^11,12^.

Disease burdens on Chinese and Japanese populations are more severe compared with the global average levels. In China, the disease burden has been increasing over the past decades^13^ with a concomitant increase in the age-standardized mortality rates (ASMRs) attributable to high SBP of at least 110–115 mmHg from 1232.6 to 2334.5 per 100,000 population during the period between 1990 and 2015^2^. In Japan, the corresponding ASMRs increased, however in a slower pace, from 189.1 to 227.3 per 100,000 population during the same period^2^. Despite the stroke burden has decreased in Japan during the previous decades, this burden is expected to increase again due to the rapid aging of the Japanese population^14^. To understand the gap in stroke burden trends between the two countries, it is crucial to investigate the possible role of increased SBP as a potential contributor to an extra reduction in stroke burden in Japan in comparison with China. We, therefore, used data from the Global Burden of Disease (GBD) 2017 to investigate the differences in stroke mortality attributable to SBP of at least 110–115 mmHg in China and Japan during the period between 1990 and 2017. Since mortality rates reflect the accumulation of health risks^15^, we further investigated the independent impacts of age, period, and cohort on the long-term trends via an age-period-cohort model (APC) of estimable functions using the same data.

## Methods

### Global burden of disease study 2017

The GBD 2017 study evaluated risk-specific and age- and sex-specific rates of several risk factors for mortality nationally, regionally, and globally during the period between 1990 and 2017^16,17^. In the GBD 2017, data on stroke mortality in China were obtained from the Chinese Center for Disease Control and Prevention Cause of Death Reporting System, the Disease Surveillance Points, and the Maternal and Child Surveillance System in the country, while data on stroke mortality in Japan were obtained from the Japanese Social Health Insurance System^18,19^. Stroke was defined per the clinical criteria of the World Health Organization^20^. High SBP was defined as SBP of at least 110–115 mmHg^21^.

### Statistical analyses

In this study, an APC framework was utilized to appraise the roles of age, period, and cohort in high SBP-attributable stroke mortality. The age effect represented the sociodemographic and biological factors affecting aging. The period effect reflected over-time changes affecting high SBP-attributable stroke mortality among all age groups such as applying preventive measures or providing new medical procedures. The cohort effect reflected the variations in high SBP-attributable stroke mortality across generations due to certain risk factors shared by each generation^22–25^. APC analysis has the following functions: (1) the net drift which assesses the annual percentage change via calculating the overall log-linear trend by cohort and period, (2) the local drifts which assesses the annual percentage changes for each age group via calculating the log-linear trend by cohort and period, (3) the longitudinal age curves which shows the fitted longitudinal age-specific rates in the reference cohort adjusted for period deviations, and (4) the cohort/period rate ratios (RR) that represents the cohort/ period relative risk, compared to the reference one, after adjustment for age and nonlinear cohort and period effects.

The study of the GBD 2017 recorded the date of death, location, sex, age (progressive 5-year age groups), year (consecutive 5-year periods from 1990), disease causes, risk factors, metric (number and rate of cases), and value (upper and lower). The SBP-attributable stroke mortality was measured based on the following four components: the number of stroke deaths, the exposure levels for SBP, the relative risk of stroke mortality due to SBP, and the counterfactual level of SBP exposure. Rates of SBP-attributable stroke mortality were calculated as the total number of stroke deaths multiplied by the population attributable fraction (PAF) for stroke-SBP pair in a given year, location, sex, and age; where the PAF identifies what percentage of stroke mortality could be avoided in a certain year if the exposure to SBP in the past were increased to the counterfactual level of the theoretical minimum risk exposure level (SBP ≤ 110–115 mmHg)^26^. The sex-specific high SBP-attributable stroke mortality rates in both countries were age-standardized according to the GBD 2013 global age-standard population and the ASMRs were calculated by the calculation formulas^18,27^. Since the period and age intervals should be fixed and equal in the APC tool, all individuals aged 80 years or older could not be analyzed in this study because they were recorded as one group only in the GBD database. SBP-attributable stroke mortality is rare below the age of 25 years, people aged < 25 years were also not included in the analyses. Central age groups, cohort, and period were defined as the reference.

The estimable functions were used to perform the APC analysis. APC Web Tool (Biostatistics Branch, National Cancer Institute, Bethesda, MD https://analysistools.nci.nih.gov/apc/) was used to get the estimable parameters^28,29^.

We compared overall net drifts, local drifts, longitudinal age curves, and cohort/period rate ratios (RRs) for high SBP-attributable stroke mortality in men and women of China and Japan. Wald chi-square tests were used to investigate the statistical significance of the estimable functions. To assess the statistical significance of the slopes for the period/cohort RRs, the interaction between sex and calendar year/birth cohort was assessed using general linear models. All statistical analyses were performed using the SAS Version 9.4 software (SAS Institute Inc, Cary, NC), and all statistical tests were two-tailed.

### Ethical statement

This study used deidentified publicly available data from the Global Burden of Disease Study 2017 repository. Thus, ethical approval and an ethical statement from an institutional review board or ethics committee were not required for the secondary analysis of data for China and Japan.

## Results

**Figure 1:**
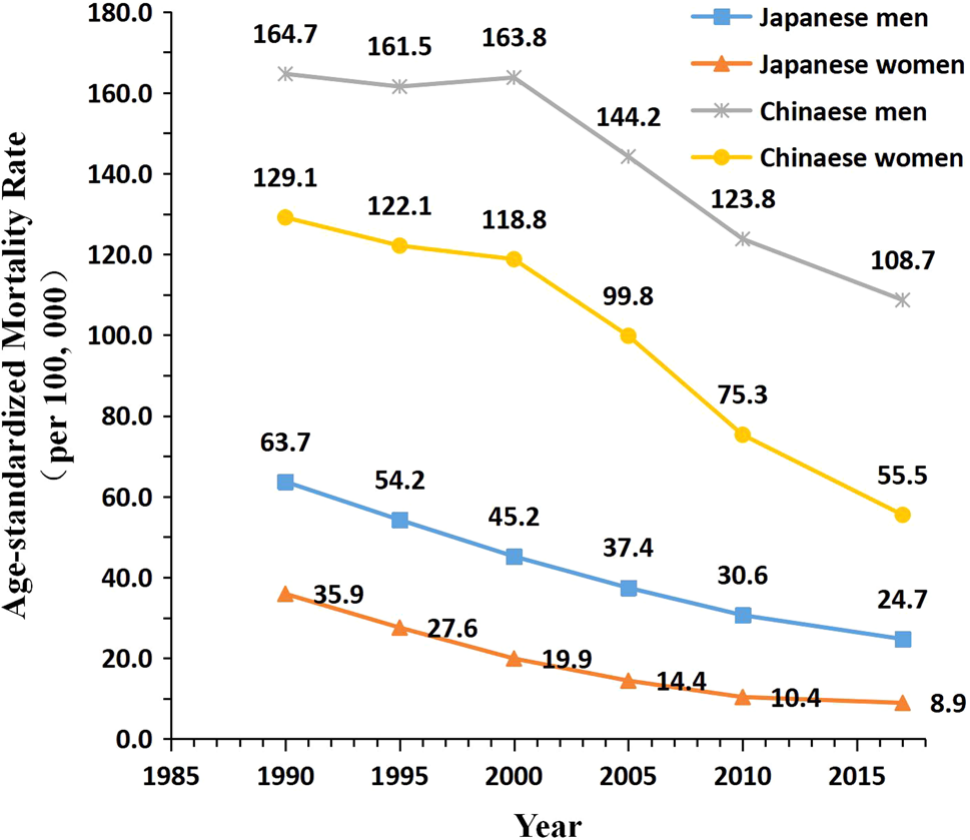
Trends of the age-standardized mortality rates (ASMR) per 100 000 population for high SBP-attributable stroke mortality by sex in China and Japan, 1990 to 2017. Standardized to the GBD 2013 (Global Burden of Disease Study 2013) global age-standard population. *Japanese men* 1990: Population: 38 067 229; Stroke: 38 690, 1995: Population: 40 061 997; Stroke: 43 172, 2000: Population: 42 325 105; Stroke: 53 896, 2005: Population: 43 537 328; Stroke: 56 710, 2010: Population: 43 9242 91; Stroke: 51 523, 2017: Population: 43 375 707; Stroke: 49 687. *Japanese women* 1990: Population: 40 384 673; Stroke: 38 284, 1995: Population: 42 106 114; Stroke: 36 083, 2000: Population: 44 166 128; Stroke: 33 924, 2005: Population: 45 113 407; Stroke: 27 271, 2010: Population: 45 222 792; Stroke: 21 438, 2017: Population: 44 420 972; Stroke: 18 971. *Chinese men* 1990: Population: 290 646 432; Stroke: 2 169 970, 1995: Population: 337 919 965; Stroke: 2 769 952, 2000: Population: 380 394 008; Stroke: 3 551 441, 2005: Population: 409 095 096; Stroke: 4 620 059, 2010: Population: 436 732 774; Stroke: 5 592 961, 2017: Population: 483 291 490; Stroke: 7 389 792. *Chinese women* 1990: Population: 275 594 048; Stroke: 1 336 893, 1995: Population: 322 776 513; Stroke: 1 643 057, 2000: Population: 365 152 409; Stroke: 2 082 019, 2005: Population: 393 674 755; Stroke: 2 545 186, 2010: Population: 421 787 480; Stroke: 2 723 681, 2017: Population: 466 391 682; Stroke: 3 082 720.

Between 1990 and 2017, in China, the high SBP-attributable stroke mortality rate per 100,000 population declined from 164.7 to 108.7 in men and from 129.1 to 55.5 in women. In Japan, the corresponding rates were 63.7 and 24.7 in men and 35.9 and 8.9 in women, respectively. The overall declining ASMR trends in China were faster than before the year 2000 (Fig. 1).

**Figure 2:**
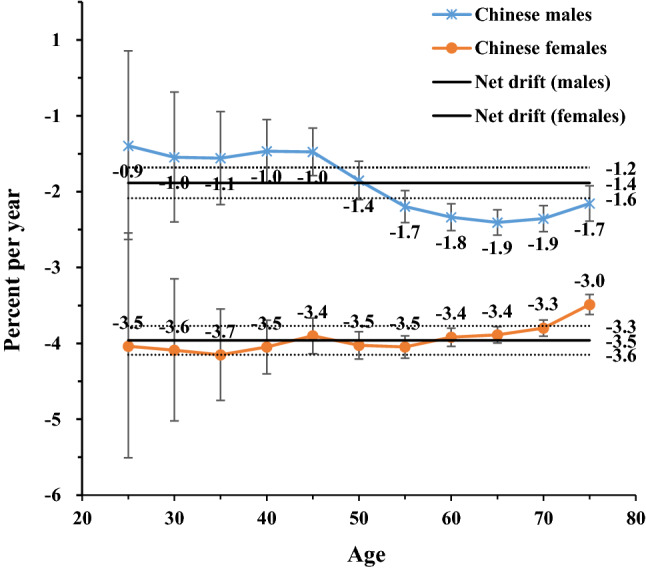
Local drift with net drift values for high SBP-attributable stroke mortality in China. Age group-specific annual percent change (local drift) with the overall annual percent change (net drift) in high sodium-attributable stroke mortality rate. Net drift values are depicted as solid lines with dashed lines representing their 95% CIs. Error bars represent the 95% CIs for the local drift values. *Japanese men* 1990: Population: 38 067 229; Stroke: 38 690, 1995: Population: 40 061 997; Stroke: 43 172, 2000: Population: 42 325 105; Stroke: 53 896, 2005: Population: 43 537 328; Stroke: 56 710, 2010: Population: 43 9242 91; Stroke: 51 523, 2017: Population: 43 375 707; Stroke: 49 687. *Japanese women* 1990: Population: 40 384 673; Stroke: 38 284, 1995: Population: 42 106 114; Stroke: 36 083, 2000: Population: 44 166 128; Stroke: 33 924, 2005: Population: 45 113 407; Stroke: 27 271, 2010: Population: 45 222 792; Stroke: 21 438, 2017: Population: 44 420 972; Stroke: 18 971. *Chinese men* 1990: Population: 290 646 432; Stroke: 2 169 970, 1995: Population: 337 919 965; Stroke: 2 769 952, 2000: Population: 380 394 008; Stroke: 3 551 441, 2005: Population: 409 095 096; Stroke: 4 620 059, 2010: Population: 436 732 774; Stroke: 5 592 961, 2017: Population: 483 291 490; Stroke: 7 389 792. *Chinese women* 1990: Population: 275 594 048; Stroke: 1 336 893, 1995: Population: 322 776 513; Stroke: 1 643 057, 2000: Population: 365 152 409; Stroke: 2 082 019, 2005: Population: 393 674 755; Stroke: 2 545 186, 2010: Population: 421 787 480; Stroke: 2 723 681, 2017: Population: 466 391 682; Stroke: 3 082 720.

**Figure 3:**
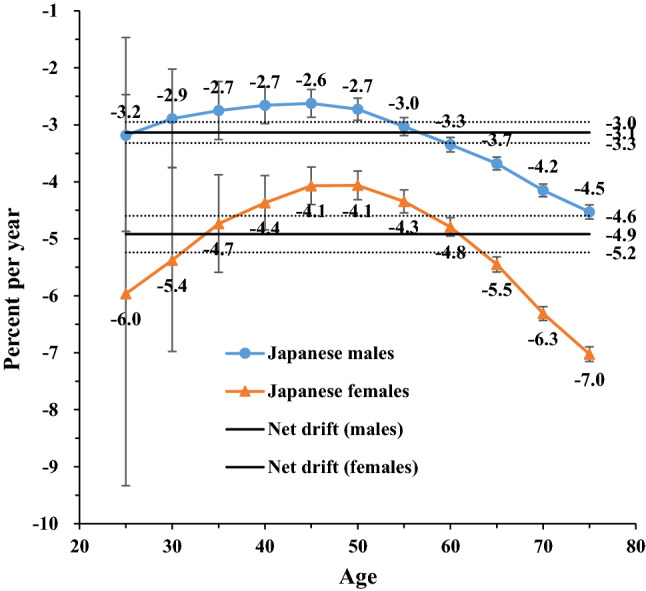
Local drift with net drift values for high SBP-attributable stroke mortality in Japan. Age group-specific annual percent change (local drift) with the overall annual percent change (net drift) in high-sodium-intake-attributable stroke mortality rate. Net drift values are depicted as solid lines with dashed lines representing their 95% CIs. Error bars represent the 95% CIs for the local drift values. *Japanese men* 1990: Population: 38 067 229; Stroke: 38 690, 1995: Population: 40 061 997; Stroke: 43 172, 2000: Population: 42 325 105; Stroke: 53 896, 2005: Population: 43 537 328; Stroke: 56 710, 2010: Population: 43 9242 91; Stroke: 51 523, 2017: Population: 43 375 707; Stroke: 49 687. *Japanese women* 1990: Population: 40 384 673; Stroke: 38 284, 1995: Population: 42 106 114; Stroke: 36 083, 2000: Population: 44 166 128; Stroke: 33 924, 2005: Population: 45 113 407; Stroke: 27 271, 2010: Population: 45 222 792; Stroke: 21 438, 2017: Population: 44 420 972; Stroke: 18 971. *Chinese men* 1990: Population: 290 646 432; Stroke: 2 169 970, 1995: Population: 337 919 965; Stroke: 2 769 952, 2000: Population: 380 394 008; Stroke: 3 551 441, 2005: Population: 409 095 096; Stroke: 4 620 059, 2010: Population: 436 732 774; Stroke: 5 592 961, 2017: Population: 483 291 490; Stroke: 7 389 792. *Chinese women* 1990: Population: 275 594 048; Stroke: 1 336 893, 1995: Population: 322 776 513; Stroke: 1 643 057, 2000: Population: 365 152 409; Stroke: 2 082 019, 2005: Population: 393 674 755; Stroke: 2 545 186, 2010: Population: 421 787 480; Stroke: 2 723 681, 2017: Population: 466 391 682; Stroke: 3 082 720.

Throughout the period between 1990 and 2017, the annual net drift values were − 1.4% in Chinese men, − 3.5% in Chinese women, − 3.1% in Japanese men, and − 4.9% in Japanese women (*p* < 0.01 for all). In both countries, the local drift values were < 0 among all age groups and were lower among women compared to men. Yet, the sex-specific local drift curves for high SBP-attributable stroke mortality showed certain trends. In China, the trends were increasing in 45 years old among Chinese men and 55–79 years old among Chinese women. In Japan, these trends increased in both sexes before 45 years old but decreased in older ages (Figs. 2, 3).

**Figure 4:**
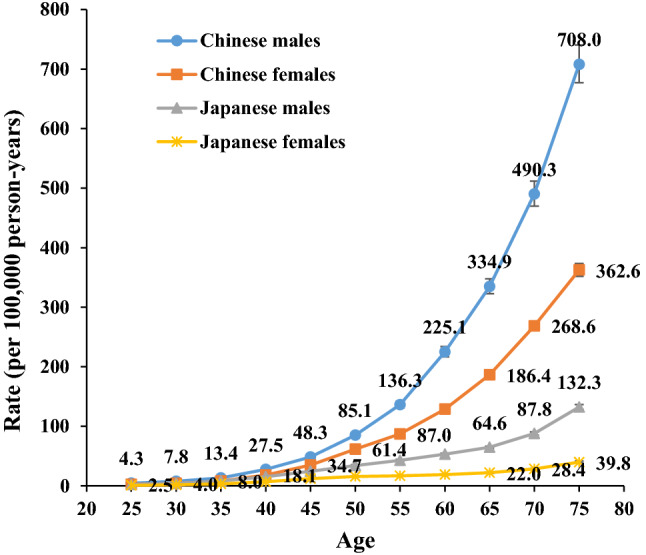
Longitudinal age curves of high SBP-attributable stroke mortality in China and Japan. Fitted longitudinal age-specific rates of high-sodium-intake-attributable stroke mortality (per 100 000 person-years). Error bars represent the 95%CIs for the Longitudinal age curve values. *Japanese men* 1990: Population: 38 067 229; Stroke: 38 690, 1995: Population: 40 061 997; Stroke: 43 172, 2000: Population: 42 325 105; Stroke: 53 896, 2005: Population: 43 537 328; Stroke: 56 710, 2010: Population: 43 9242 91; Stroke: 51 523, 2017: Population: 43 375 707; Stroke: 49 687. *Japanese women* 1990: Population: 40 384 673; Stroke: 38 284, 1995: Population: 42 106 114; Stroke: 36 083, 2000: Population: 44 166 128; Stroke: 33 924, 2005: Population: 45 113 407; Stroke: 27 271, 2010: Population: 45 222 792; Stroke: 21 438, 2017: Population: 44 420 972; Stroke: 18 971. *Chinese men* 1990: Population: 290 646 432; Stroke: 2 169 970, 1995: Population: 337 919 965; Stroke: 2 769 952, 2000: Population: 380 394 008; Stroke: 3 551 441, 2005: Population: 409 095 096; Stroke: 4 620 059, 2010: Population: 436 732 774; Stroke: 5 592 961, 2017: Population: 483 291 490; Stroke: 7 389 792. *Chinese women* 1990: Population: 275 594 048; Stroke: 1 336 893, 1995: Population: 322 776 513; Stroke: 1 643 057, 2000: Population: 365 152 409; Stroke: 2 082 019, 2005: Population: 393 674 755; Stroke: 2 545 186, 2010: Population: 421 787 480; Stroke: 2 723 681, 2017: Population: 466 391 682; Stroke: 3 082 720.

The peak mortality rates per 100,000 person-years reached 708.0 in Chinese men, 362.6 in Chinese women, 132.3 in Japanese men, and 39.8 in Japanese women. The high SBP-attributable stroke risk, in both countries, was higher among men than among women across all age groups (Fig. 4).

**Figure 5:**
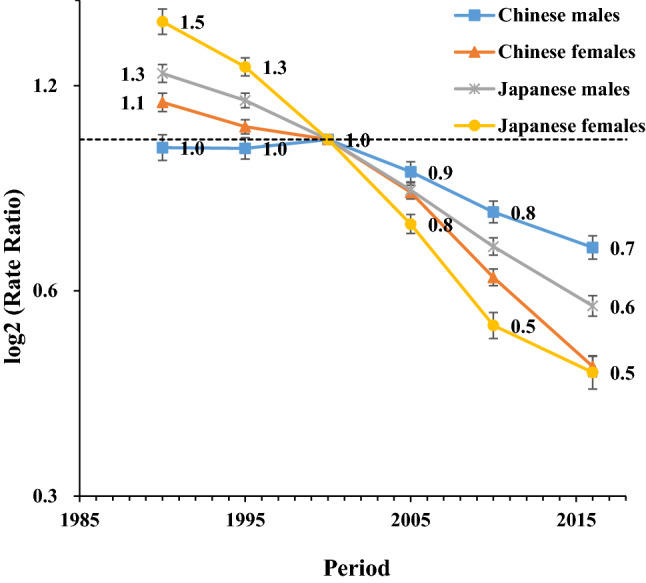
Period relative risks (RRs) of high SBP-attributable stroke mortality rate by sex in China and Japan. The relative risk of each period compared with the reference one (the year 2000) adjusted for age and nonlinear cohort effects. Error bars represent the 95%CIs for the period relative risks. *Japanese men* 1990: Population: 38 067 229; Stroke: 38 690, 1995: Population: 40 061 997; Stroke: 43 172, 2000: Population: 42 325 105; Stroke: 53 896, 2005: Population: 43 537 328; Stroke: 56 710, 2010: Population: 43 9242 91; Stroke: 51 523, 2017: Population: 43 375 707; Stroke: 49 687. *Japanese women* 1990: Population: 40 384 673; Stroke: 38 284, 1995: Population: 42 106 114; Stroke: 36 083, 2000: Population: 44 166 128; Stroke: 33 924, 2005: Population: 45 113 407; Stroke: 27 271, 2010: Population: 45 222 792; Stroke: 21 438, 2017: Population: 44 420 972; Stroke: 18 971. *Chinese men* 1990: Population: 290 646 432; Stroke: 2 169 970, 1995: Population: 337 919 965; Stroke: 2 769 952, 2000: Population: 380 394 008; Stroke: 3 551 441, 2005: Population: 409 095 096; Stroke: 4 620 059, 2010: Population: 436 732 774; Stroke: 5 592 961, 2017: Population: 483 291 490; Stroke: 7 389 792. *Chinese women* 1990: Population: 275 594 048; Stroke: 1 336 893, 1995: Population: 322 776 513; Stroke: 1 643 057, 2000: Population: 365 152 409; Stroke: 2 082 019, 2005: Population: 393 674 755; Stroke: 2 545 186, 2010: Population: 421 787 480; Stroke: 2 723 681, 2017: Population: 466 391 682; Stroke: 3 082 720.

**Figure 6:**
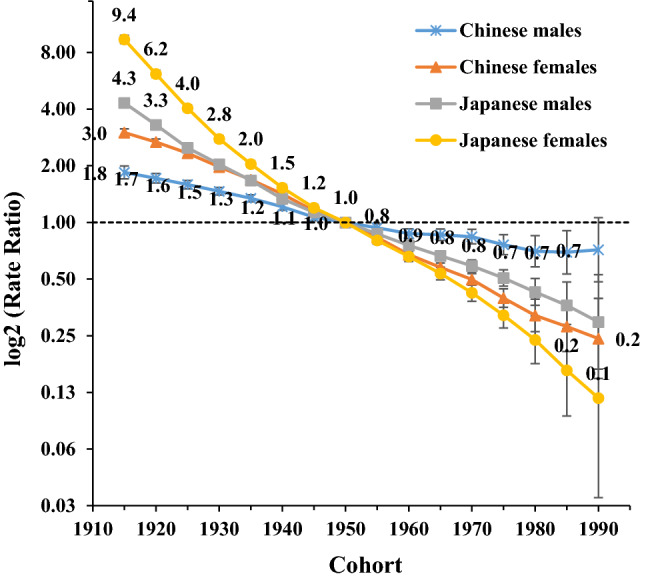
Cohort relative risks (RRs) of high SBP-attributable stroke mortality rate by sex in China and Japan. The relative risk of each cohort compared with the reference one (cohort 1950) adjusted for age and nonlinear period effects. Error bars represent the 95%CIs for the cohort relative risks. *Japanese men* 1990: Population: 38 067 229; Stroke: 38 690, 1995: Population: 40 061 997; Stroke: 43 172, 2000: Population: 42 325 105; Stroke: 53 896, 2005: Population: 43 537 328; Stroke: 56 710, 2010: Population: 43 9242 91; Stroke: 51 523, 2017: Population: 43 375 707; Stroke: 49 687. *Japanese women* 1990: Population: 40 384 673; Stroke: 38 284, 1995: Population: 42 106 114; Stroke: 36 083, 2000: Population: 44 166 128; Stroke: 33 924, 2005: Population: 45 113 407; Stroke: 27 271, 2010: Population: 45 222 792; Stroke: 21 438, 2017: Population: 44 420 972; Stroke: 18 971. *Chinese men* 1990: Population: 290 646 432; Stroke: 2 169 970, 1995: Population: 337 919 965; Stroke: 2 769 952, 2000: Population: 380 394 008; Stroke: 3 551 441, 2005: Population: 409 095 096; Stroke: 4 620 059, 2010: Population: 436 732 774; Stroke: 5 592 961, 2017: Population: 483 291 490; Stroke: 7 389 792. *Chinese women* 1990: Population: 275 594 048; Stroke: 1 336 893, 1995: Population: 322 776 513; Stroke: 1 643 057, 2000: Population: 365 152 409; Stroke: 2 082 019, 2005: Population: 393 674 755; Stroke: 2 545 186, 2010: Population: 421 787 480; Stroke: 2 723 681, 2017: Population: 466 391 682; Stroke: 3 082 720.

The period RRs among men and women showed declining trends in both countries (except for Chinese men in 1990–2000), with a relatively more rapid decline in women compared with men and in Japanese people compared with Chinese people. Despite Chinese people had lower high SBP-attributable stroke mortality than Japanese people during the 1990s, RRs crossed in the year 2000 before decreasing at a slower pace among Chinese people in comparison with Japanese people. Alike, the Chinese and Japanese cohort RRs crossed in the year 1950, with greater high SBP-attributable stroke mortality rates before then among Japanese people, but lower rates afterward in comparison with Chinese people. The cohort RRs in both countries showed declining trends among men and women with a more significant decline in women than men across all birth cohorts (Figs. 5, 6). The cohort and period RRs in both sexes were statistically significant whether in China or Japan (*p* < 0.01) in addition to the net and local drifts except for the local drift in Japanese men (Table 1).

**Table 1:** Wald Chi-Square tests for estimable functions in the APC model.

Null hypothesis	China				Japan			
	Men		Women		Men		Women	
	*χ*^2^	*P-*Value	*χ*^2^	*P*-Value	*χ*^2^	*P*-Value	*χ*^2^	*P*-Value
Net Drift = 0	177.1	< 0.001	1238.4	< 0.001	1074.2	< 0.001	855.2	< 0.001
All Period RR = 1	372.1	< 0.001	2188.6	< 0.001	1208.2	< 0.001	981.9	< 0.001
All Cohort RR = 1	856.6	< 0.001	7362.0	< 0.001	9162.8	< 0.001	16,298.3	< 0.001
All Local Drifts = Net Drift	32.1	< 0.001	37.5	< 0.001	302.9	< 0.001	671.4	< 0.001

## Discussion

This study indicated that, between 1990 and 2017, the ASMRs and cohort/period RRs for high SBP-attributable stroke mortality showed declining trends among Chinese and Japanese men and women from all age groups. Yet, high SBP-attributable stroke mortality longitudinal age curves grew rapidly among Chinese men and women compared with their Japanese counterparts.

The declining trends of high SBP-attributable stroke mortality in China and Japan reflect the improvement of high SBP control in both countries. Such control could not have been achieved in both countries without the adherence of their people to healthy behaviors such as physical activity and salt restriction in addition to the significant signs of progress in health indicators such as healthcare coverage and advancement of the clinical diagnosis and treatment technology. These factors might have contributed together to the SBP control and consequently the declining trends of stroke in China and Japan^30–36^.

Of note, this study showed that high SBP-attributable stroke mortality was higher among men than women and older than younger people. Age is, by far, the topmost demographic risk factor for stroke. A prospective cohort study on a half million adults in China showed that SBP increased linearly with age in both sexes and was log-linearly associated with stroke risk. Every 10 mmHg higher of usual SBP was associated with a 30% higher risk of ischemic stroke and 68% increased risk of intracerebral hemorrhage, with men more vulnerable than women at every age group^37^.

It is worth pointing out that this study had two major strengths, First, it is the first study to investigate the impacts of age-period-cohort on the temporal trends of stroke mortality attributable to high SBP in China and Japan. Second, GBD 2017 provided estimates of cause-specific and age- and sex-specific mortality rates that were internally consistent, thus, the possibility of misclassification bias was unlikely. However, some limitations should be addressed. First, because the GBD data was generated by systematic reviewing and synthesizing of published and unpublished data, there are many uncertainty issues related to measurement errors, missing data, and systematic bias of the countries’ prior estimates of the risk factors and outcomes pooled in the GBD^38^ which could add bias to the cross-national comparisons. This is truly right for countries with poor vital registration systems. However, we compared stroke mortality, in Japan and China, where the death certificate diagnosis of stroke was highly valid due to the widespread use of CT and MRI in both countries since the 1980s^39,40^. Also, the GBD data estimated SBP for the year, sex, and age group, and was a more comprehensive system of risk analysis than any previous global or national analysis which could assess the changes in SBP burden^41^. Second, since period and age intervals have to be fixed and equal in the APC tool, those who were aged ≥ 80 years were excluded from this study because they were merged in one group only in the GBD database. However, the stroke mortality rate among people aged ≥ 80 years was shown to decline in a similar pattern^42^. Another, we did not include people aged ≤ 25 years as stroke mortality attributable to high SBP is considered negligible in this age group. Third, we could not stratify our results by stroke subtype because of the absence of data on the Japanese stroke subtype. Fourth, one main limitation of the APC analyses is that it could be conducted only on the pair of a risk factor and an outcome (high SBP and stroke ASMR in this research). Many other risk factors, such as alcohol intake, tobacco smoking, and even clinical advancement contribute to the ASMR of stroke and may substantially affect high SBP-attributable stroke ASMR because these factors are related to both the blood pressure and the stroke mortality, adding to the above-mentioned uncertainties.

In conclusion, the ASMRs, as well as the period and cohort RRs of high SBP-attributable stroke mortality, declined in China and Japan in both sexes and across all age groups during the period between 1990 to 2017. However, the proportion of the high SBP population remained significant in both countries. Thus, policymakers, especially in China, should prioritize hypertension control approaches to reduce the future risk of stroke mortality.

## References

[1] Lim SS (2012). A comparative risk assessment of burden of disease and injury attributable to 67 risk factors and risk factor clusters in 21 regions, 1990–2010: A systematic analysis for the Global Burden of Disease Study 2010. Lancet.

[2] Forouzanfar MH (2017). Global burden of hypertension and systolic blood pressure of at least 110 to 115 mm Hg, 1990–2015. JAMA.

[3] Inoue R (2009). Stroke risk of blood pressure indices determined by home blood pressure measurement: The Ohasama Study. Stroke.

[4] Psaty BM (2001). Association between blood pressure level and the risk of myocardial infarction, stroke, and total mortality: The cardiovascular health study. Arch. Int. Med..

[5] Asia Pacific Cohort Studies Collaboration (2003). Blood pressure indices and cardiovascular disease in the Asia Pacific region: A Pooled analysis. Hypertension.

[6] Lawes CMM, Vander HS, Rodgers A (2008). For the International Society of Hypertension: Global burden of blood-pressure-related disease, 2001. Lancet.

[7] Lawes CM (2006). Blood pressure and the global burden of disease 2000: Part II: Estimates of attributable burden. J. Hypertens..

[8] Kernan WN (2014). Guidelines for the prevention of stroke in patients with stroke and transient ischemic attack: A guideline for healthcare professionals from the American Heart Association/American Stroke Association. Stroke.

[9] Lewington S, Clarke R, Qizilbash N, Prospective Studies Collaboration (2002). Age-specific relevance of usual blood pressure to vascular mortality: A meta-analysis of individual data for one million adults in 61 prospective studies. Lancet.

[10] Lawes CM (2004). Blood pressure and stroke: An overview of published reviews. Stroke.

[11] Johansson BB (1999). Hypertension mechanisms causing stroke. Clin. Exp. Pharmacol. Physiol..

[12] Yu JG, Zhou RR, Cai GJ (2011). From hypertension to stroke: Mechanisms and potential prevention strategies. CNS Neurosci. Ther..

[13] Wang W (2017). Prevalence, incidence, and mortality of stroke in china: Results from a nationwide population-based survey of 480 687 adults. Circulation.

[14] Takashima N (2017). Incidence, management and short-term outcome of stroke in a general population of 1.4 million Japanese- Shiga Stroke Registry. Circ. J..

[15] Wang J (2017). Comparison of secular trends in cervical cancer mortality in China and the United States: An Age-Period-Cohort Analysis. Int. J. Environ. Res. Public Health.

[16] Gakidou E (2017). Global, regional, and national comparative risk assessment of 84 behavioural, environmental and occupational, and metabolic risks or clusters of risks, 1990–2017: A systematic analysis for the Global Burden of Disease Study 2017. Lancet.

[17] GBD 2017 Causes of Death Collaborators (2017). Global, regional, and national age-sex specific mortality for 264 causes of death, 1980–2017: A systematic analysis for the Global Burden of Disease Study 2017. Lancet.

[18] Zhou M (2017). Cause-specific mortality for 240 causes in China during 1990–2013: A systematic subnational analysis for the Global Burden of Disease Study 2013. Lancet.

[19] Nomura S (2017). Population health and regional variations of disease burden in Japan, 1990–2015: A systematic subnational analysis for the Global Burden of Disease Study 2015. Lancet.

[20] Aho K (1980). Cerebrovascular disease in the community: Results of a WHO collaborative study. Bull. World Health Organ..

[21] GBD 2015 risk factors collaborators (2017). Global, regional, and national comparative risk assessment of 79 behavioural, environmental and occupational, and metabolic risks or clusters of risks, 1990–2015: A systematic analysis for the Global Burden of Disease Study 2015. Lancet.

[22] Holford TR (1983). The estimation of age, period and cohort effects for vital rates. Biometrics.

[23] Tango T, Kurashina S (1987). Age, period and cohort analysis of trends in mortality from major diseases in Japan, 1955 to 1979: Peculiarity of the cohort born in the early Showa Era. Stat. Med..

[24] Medrano MJ (1997). Effect of age, birth cohort, and period of death on cerebrovascular mortality in Spain, 1952 through 1991. Stroke.

[25] Peltonen M, Asplund K (1996). Age-period-cohort effects on stroke mortality in Sweden 1969–1993 and forecasts up to the year 2003. Stroke.

[26] GBD 2016 Causes of Death Collaborators (2017). Global, regional, and national age-sex specific mortality for 264 causes of death, 1980–2016: A systematic analysis for the Global Burden of Disease Study 2016. Lancet.

[27] Cao J, Eshak ES, Yang J, Liu K, Geo K, Liu Z, Yu C (2020). Age-period-cohort analysis of stroke mortality attributable to low physical activity in China and Japan: Data from the Global Burden of Disease Study 1990–2016. Sci. Rep..

[28] Rosenberg PS, Check DP, Anderson WF (2014). A web tool for age-period-cohort analysis of cancer incidence and mortality rates. Cancer Epidemiol. Biomarkers Prev..

[29] Tu YK, Krämer N, Lee WC (2012). Addressing the identification problem in age-period-cohort analysis: A tutorial on the use of partial least squares and principal components analysis. Epidemiology.

[30] Ministry of Health, Labour and Welfare. National Health and Nutrition Examination Survey, (1990). http://www.nibiohn.go.jp/eiken/kenkounippon21/eiyouchousa/kekka_shintai_chousa_nendo.html

[31] China Health and Nutrition Survey. https://www.cpc.unc.edu/projects/china

[32] Chen XR (2012). Leisure-time physical activity and sedentary behaviors among Chinese adults in 2010. Chin. J. Prev. Med..

[33] Li H (2017). The development and impact of primary health care in China from 1949 to 2015: A focused review. Int. J. Health Plan. Manag..

[34] Zhang X, Oyama T (2016). Investigating the health care delivery system in Japan and reviewing the local public hospital reform. Risk Manag. Healthcare Policy.

[35] Wang W (2017). Trend of declining stroke mortality in China: Reasons and analysis. Stroke Vasc. Neurol..

[36] Imano H (2009). Trends for blood pressure and its contribution to stroke incidence in the middle-aged Japanese population: The Circulatory Risk in Communities Study (CIRCS). Stroke.

[37] Lacey B (2018). Age-specific association between blood pressure and vascular and non-vascular chronic diseases in 0.5 million adults in China: A prospective cohort study. Lancet Glob. Health.

[38] Morfeld P, Erren TC (2019). Uncertainties in the GBD 2017 estimates on diet and health. Lancet.

[39] Iso H, Jacobs DR, Goldman L (1990). Accuracy of death certificate diagnosis of intracranial hemorrhage and nonhemorrhagic stroke: The Minnesota Heart Survey. Am. J. Epidemiol..

[40] Yang G (2005). Mortality registration and surveillance in China: History, current situation and challenges. Popul. Health Metrics.

[41] Lim SS (2012). A comparative risk assessment of burden of disease and injury attributable to 67 risk factors and risk factor clusters in 21 regions, 1990–2010: A systematic analysis for the Global Burden of Disease Study 2010. Lancet.

[42] Wang Z (2017). Age-period-cohort analysis of stroke mortality in China: Data from the global burden of disease study 2013. Stroke.

